# Urinary Macrophage Migration Inhibitory Factor Serves as a Potential Biomarker for Acute Kidney Injury in Patients with Acute Pyelonephritis

**DOI:** 10.1155/2012/381358

**Published:** 2012-12-23

**Authors:** Ming-Yuan Hong, Chin-Chung Tseng, Chia-Chang Chuang, Chia-Ling Chen, Sheng-Hsiang Lin, Chiou-Feng Lin

**Affiliations:** ^1^Institute of Clinical Medicine, College of Medicine, National Cheng Kung University, Tainan 701, Taiwan; ^2^Department of Emergency Medicine, National Cheng Kung University Hospital, Tainan 704, Taiwan; ^3^Department of Internal Medicine, National Cheng Kung University Hospital, Tainan 704, Taiwan; ^4^Department of Microbiology and Immunology, College of Medicine, National Cheng Kung University, Tainan 701, Taiwan; ^5^Research Center of Clinical Medicine, College of Medicine, National Cheng Kung University, Tainan 701, Taiwan; ^6^Institute of Basic Medical Sciences, College of Medicine, National Cheng Kung University, Tainan 701, Taiwan; ^7^Center of Infectious Disease and Signaling Research, College of Medicine, National Cheng Kung University, Tainan 701, Taiwan

## Abstract

Conventional markers of kidney function that are familiar to clinicians, including the serum creatinine and blood urea nitrogen levels, are unable to reveal genuine injury to the kidney, and their use may delay treatment. Macrophage migration inhibitory factor (MIF) is a proinflammatory cytokine, and the predictive role and pathogenic mechanism of MIF deregulation during kidney infections involving acute kidney injury (AKI) are not currently known. In this study, we showed that elevated urinary MIF levels accompanied the development of AKI during kidney infection in patients with acute pyelonephritis (APN). In addition to the MIF level, the urinary levels of interleukin (IL)-1**β** and kidney injury molecule (KIM)-1 were also upregulated and were positively correlated with the levels of urinary MIF. An elevated urinary MIF level, along with elevated IL-1**β** and KIM-1 levels, is speculated to be a potential biomarker for the presence of AKI in APN patients.

## 1. Introduction

Even minor increases in the serum creatinine level are associated with an increased risk of inhospital morbidity and mortality [1]. A modest decline in the glomerular filtration rate and kidney injury should be used to diagnose kidney damage to facilitate early detection and intervention [2, 3]. Therefore, the RIFLE (risk, injury, failure, loss, and end-stage kidney disease) criteria replace the term “acute renal failure” with “acute kidney injury” (AKI) [4]. However, traditional tools, including the serum creatinine and blood urea nitrogen (BUN) levels and urinary markers (urinary output and urine sodium excretion) are not sufficiently sensitive to provide an early diagnosis of AKI, and their use may delay treatment [5, 6]. It is expected that injury biomarkers, in addition to the functional markers, will facilitate the early detection of renal injury.

Macrophage migration inhibitory factor (MIF) is a potent proinflammatory cytokine that activates macrophages and promotes the synthesis of cytokines, including tumor necrosis factor-*α*, interleukin (IL)-1*β*, and IL-8 [7, 8]. MIF is released from an intracellular pool in response to pathological stimuli including infection and inflammatory activation. MIF has been shown to bind to CD74 and recruits CD44 to form a receptor complex, resulting in the phosphorylation of extracellular signal-regulated kinase through Src tyrosine kinase [8–10]. MIF also activates transcription factors of the E-twenty-six family and upregulates Toll-like receptor (TLR) 4 expression and signaling to enhance the inflammatory response [11–14]. Renal MIF is a constitutive expression in normal kidneys and is upregulated in patients with glomerulonephritis and renal allograft rejection. The upregulation of MIF is associated with leukocyte infiltration, histopathological damage, and renal dysfunction in patients with inflammatory kidney disease [15–20]. The concentration of urinary MIF is significantly correlated with the upregulation of renal MIF expression, instead of the serum MIF level, suggesting that the elevated level of urinary MIF is due to MIF production in and secretion by the injured kidney [17, 18]. The association between the urinary MIF level and renal damage makes MIF a candidate marker for renal injury in humans.

Acute pyelonephritis (APN) is a common infection in which bacteria invade the renal epithelial cells and is an important cause of renal insufficiency [21]. Although elevated levels of urinary MIF are found in individuals with urinary tract infections (UTIs), the upregulation of MIF in the context of a UTI has only been found in patents with APN [22–24]. The correlation between the urinary MIF level and significant renal dysfunction has not yet been defined. Therefore, the aim of this study was to investigate the ability of aberrant urinary MIF levels to detect AKI in patients with kidney infections.

## 2. Materials and Methods

### 2.1. Subjects and Study Design

 Patient serum and urine samples were prospectively collected between January 2010 and December 2010 in the emergency department of the National Cheng Kung University Hospital, Tainan, Taiwan. The diagnostic criteria for APN included fever (body temperature above 38.3°C), flank pain and/or costovertebral angle tenderness with or without painful micturition, and pyuria. Thirty-nine patients who were diagnosed with symptomatic and culture-proven APN were enrolled in the study. Patients who presented with shock, urinary tract malignancy, or glomerulonephritis were excluded from the study. Patients with serum creatinine levels more than 50% above baseline were defined as having AKI according to the criteria of RIFLE [25]. The estimated glomerular filtration rate (eGFR) was calculated using the modification of diet in renal disease (MDRD) formula relative to the serum creatinine level based on age, race, and sex [26]. The blood samples for the laboratory analysis, which included a hemogram and analyses of the current renal function, C-reactive protein level, and serum MIF level, were collected within 2 hours of hospital arrival. Data including demographic information, data on comorbidities, clinical features (including blood pressure, oxygen saturation, respiratory rate, and consciousness levels, which were necessary for calculating severity scores), and baseline renal function were collected from the patients' medical records. Receiver operating curve (ROC) analysis was used to determine the ability of the urinary MIF level to predict AKI. To investigate the ability of MIF to distinguish AKI from chronic kidney disease, we also conducted a subgroup analysis including APN patients with renal dysfunction (eGFR < 60 mL/min/1.73 m^2^). The severity scores, including the Rapid Emergency Medicine Score (REMS) and the Rapid Acute Physiology Score (RAPS), were used as a measure of initial patient care [27, 28]. The REMS and RAPS scoring systems are truncated versions of the Acute Physiology and Chronic Health Evaluation (APACHEII), and they were calculated at the time of the patient's arrival at the hospital. We also included patients without UTIs as control subjects. The protocols and procedures were approved by the institutional review board of the National Cheng Kung University Hospital, Tainan, Taiwan.

### 2.2. Measurement of the Urinary Levels of MIF, IL-1*β*, and Kidney Injury Molecule (KIM)-1

Serum and urine samples were collected from APN patients and normal controls. The levels of MIF, IL-1*β*, and KIM-1 were measured using standard enzyme-linked immunosorbent assay (ELISA) kits (R&D Systems, Minneapolis, MN, USA) according to the manufacturer's recommendations. All measurements were performed in triplicate. After the reaction, the plates were washed, and 100 *μ*L of *o*-phenylenediamine substrate was added to each well. The plates were incubated for 30 min at room temperature, after which 50 *μ*L of 4 N sulfuric acid was added to each well. The plates were read at 450 nm using a microplate reader (SpectraMax 340PC; Molecular Devices, Inc., Sunnyvale, CA), and the data were analyzed (using Softmax Pro software). The levels of urinary cytokines were calculated as ratios relative to the urinary creatinine level.

### 2.3. Statistical Analysis

Values are expressed as the means ± SD. Groups were compared using Student's two-tailed unpaired *t*-test or one-way analysis of variance using SPSS 17.0 (SPSS, IBM, West Grove, USA). Pearson correlation coefficients were used to analyze the correlations between the urinary MIF level, IL-1*β* level, KIM-1 level, and white blood cell (WBC) count. A value of *P* < 0.05 was considered to be statistically significant. A receiver operating characteristic curve was used to analyze the ability to diagnose AKI based on several parameters, and the area under the curve (AUC) for each parameter was determined.

## 3. Results

### 3.1. There Is an Increase in Urinary MIF Levels in APN Patients with AKI

 To determine the clinical implications of urinary MIF in patients with kidney infections, cytokine levels and renal biochemical parameters were analyzed in patients with APN. Thirty-nine APN patients were enrolled in our study. Based on the RIFLE criteria [25], the patients were divided into two groups according to the presence of AKI. The two groups, which included 13 patients with AKI and 26 without AKI, did not differ significantly with respect to age, gender, comorbidities, laboratory data, disease severity scores, or serum MIF levels except urinary MIF levels and renal function (present BUN, creatinine, and eGFR), as shown in Table 1. The patients with AKI had an increase in urinary MIF compared to patients without AKI (17.0 ± 13.2 ng/mg versus 4.2 ± 3.5 ng/mg, *P* = 0.004). According to the power analysis for a two-group independent sample *t*-test, a sample size of 39 subjects had a reasonable power (0.97) to distinguish the two groups based on urinary MIF expression. The Gram-negative bacteria are common pathogens of UTI, including strains of *Escherichia coli, Klebsiella* spp., and *Proteus *spp. Among them, *E. coli* accounts for the 70–95% of community-acquired UTI. The microbiological analysis of the invaded pathogens was shown in Table 2. The majority of invaded pathogens in the APN patients were Gram-negative bacteria (92%), and the percentage of Gram-negative bacteria was consistent between the two groups (92%, *P* = 1.000, Table 1). To adjust the bacterial factor in altering urinary MIF expression, subgroup analysis of patients whose pathogens were identified as Gram-negative bacteria or *E. coli* was conducted in Table 3. In APN patients, invaded pathogens were identified as Gram-negative bacteria, and the urinary MIF was higher in patients with AKI compared to patients without AKI (*n* = 36, 16.5 ng/mg ± 13.8 ng/mg versus*  *4.4 ng/mg ± 3.5 ng/mg, *P* = 0.011). In patients whose invaded pathogens were proven as *E. coli*, there was consistently an increase in urinary MIF in APN patients with AKI compared to those without AKI (*n* = 27, 15.2 ng/mg ± 11.6 ng/mg versus 4.0 ng/mg ± 3.4 ng/mg, *P* = 0.013). MIF has been reported to increase and participate in the pathogenesis of diabetic nephropathy [29]. Because it remains unclear whether diabetes confounds the function of urinary MIF in detecting AKI, we analyzed the level of urinary MIF in diabetic patients (Table 3). There was an increase in urinary MIF levels in diabetic patients with AKI compared to patients without AKI (*n* = 19, 15.2 ± 8.7 ng/mg *versus* 4.1 ± 4.1 ng/mg, *P* = 0.024). 

### 3.2. The Urinary Levels of MIF, IL-1*β*, and KIM-1 Are Elevated in APN Patients with AKI

**Figure 1:**
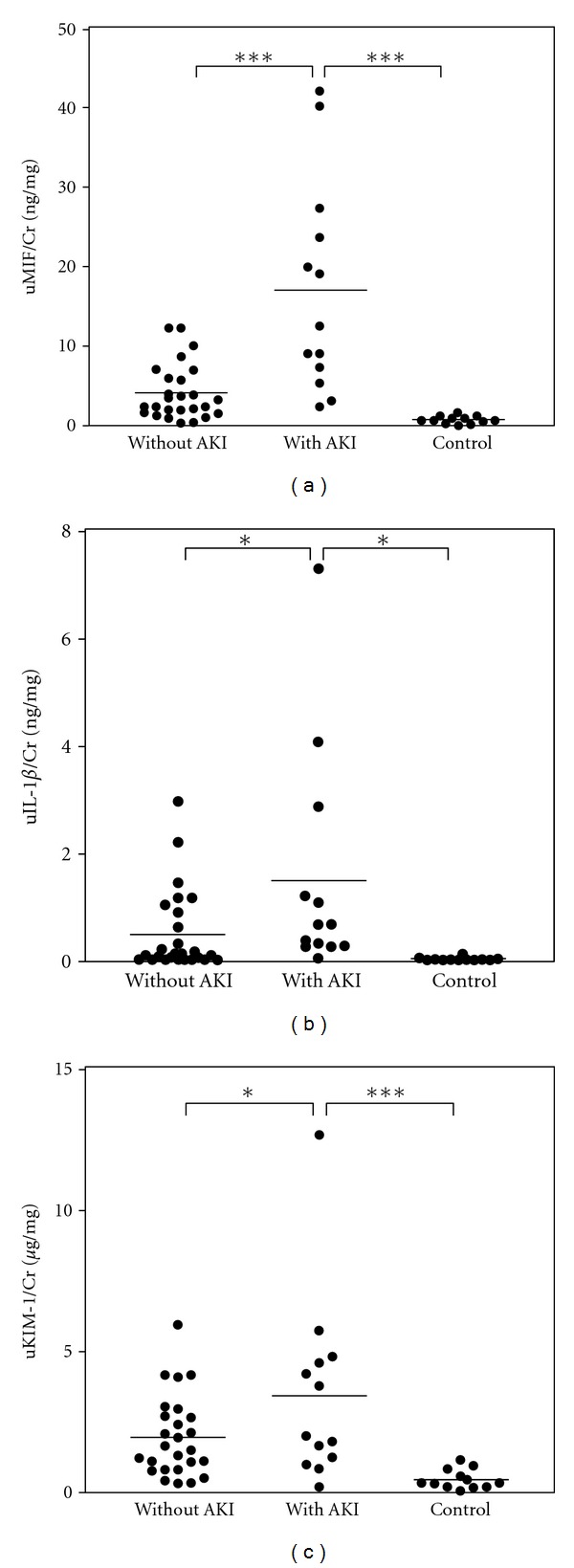
The urinary MIF level is markedly elevated in the presence of AKI in patients with kidney infections. The concentrations of MIF, IL-1*β*, and KIM-1 in the urine were measured by ELISA and were normalized based on the urinary creatinine (Cr) levels. The urinary levels of MIF (uMIF/Cr) (a), IL-1*β* (uIL-1*β*/Cr) (b), and KIM-1(uKIM-1/Cr) (c) were measured in APN patients with (*n* = 13) or without (*n* = 26) AKI and in normal controls (*n* = 12). Data are the means SD.  ***P < 0.001, and  *P < 0.05.

The urinary IL-1*β* level has been reported to be elevated in patients with APN [30], and urinary KIM-1 is a sensitive biomarker for AKI and is not influenced by UTIs or chronic kidney disease [31, 32]. We therefore evaluated the diagnostic utility of the urinary MIF, IL-1*β*, and KIM-1 levels as biomarkers for AKI during kidney infection. The levels of urinary MIF (Figure 1(a)), IL1*β* (Figure 1(b)), and KIM-1 (Figure 1(c)) were significantly higher in APN patients with AKI than in patients without AKI or normal controls. 

### 3.3. The Urinary Levels of MIF Were Positively Correlated with the Urinary Levels of IL-1*β* and KIM-1

**Figure 2:**
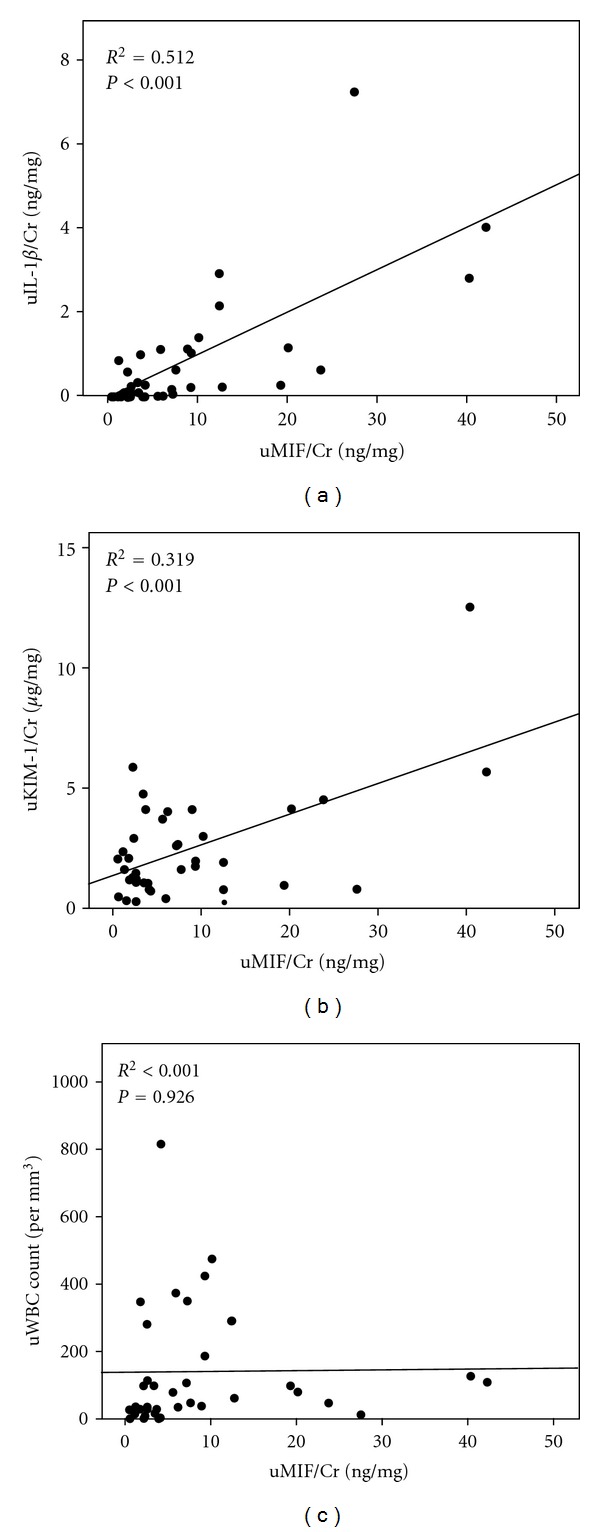
The pairwise correlations between the urinary levels of MIF (uMIF/Cr) and the urinary level of IL-1*β* (uIL-1*β*/Cr), the urinary level of KIM-1 (uKIM-1/Cr) and the urinary WBC (uWBC) count. Pearson correlation coefficients were used to analyze the correlation between two variables. Each circle represents a single individual, and lines represent linear approximations.

 The Pearson correlation coefficient was used to analyze the urinary levels of MIF, IL-1*β*, and KIM-1 and the urinary WBC count. The level of urinary MIF was positively correlated with urinary levels of IL-1*β* (*R* ^2^ = 0.512, *P* < 0.001, Figure 2(a)) and KIM-1 (*R* ^2^ = 0.319, *P* < 0.001, Figure 2(b)). However, no correlation was found between the urinary level of MIF and the urinary WBC count (*R* ^2^ < 0.001, *P* = 0.926, Figure 2(c)).

### 3.4. The AUC of the Urinary Levels of MIF for Detecting AKI among APN Patients

**Figure 3:**
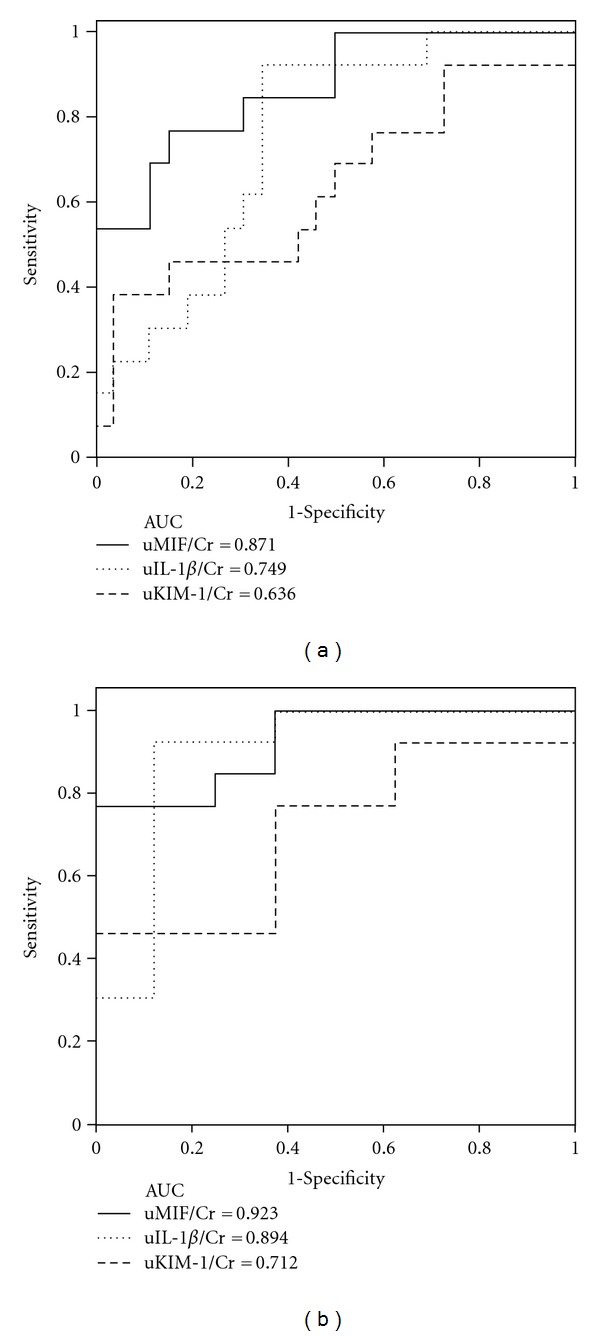
Urinary levels of MIF detected the presence of AKI in APN patients. The receiver operating characteristic curves for the laboratory parameters for the detection of AKI in all APN patients (*n* = 39) (a) or in the subgroup with renal dysfunction (eGFR < 60 ml/min/1.73 cm^2^, *n* = 21) (b). The AUCs of the urinary MIF, IL-1*β*, and KIM-1 levels, which were normalized based on the urinary creatinine (Cr) levels, are shown.

The ROC curve for detecting the presence of AKI in APN patients included the urinary levels of MIF, IL-1*β*, and KIM-1. The AUC for the urinary MIF level reached 0.871 in all APN patients (Figure 3(a)). In patient with normal renal function on arrival (defined as eGFR ≥60 mI/min/1.73 cm^2^, *n* = 18), our result revealed an elevated urinary MIF indicating the presence of kidney infection compared to normal controls (4.7 ± 3.8 ng/mg versus 0.7 ± 0.5 ng/mg,  *P* < 0.001). Encountering patients with abnormal renal function test, it is doubtable to determine whether patients have AKI or preexisting chronic kidney disease (CKD). We, therefore, analyzed the enrolled patients who presented with renal dysfunction (defined as eGFR<60 mI/min/1.73 cm^2^ on arriving at our emergency department, *n* = 21). These patients with AKI had an increase in urinary MIF compared to patients without AKI (17.0 ± 13.2 ng/mg *versus* 2.9 ± 2.4 ng/mg,  *P* = 0.002,) in Table 3. The AUC for the urinary MIF level in detecting the presence of AKI reached 0.923 in patients with renal dysfunction (Figure 3(b)). Urinary MIF helps to distinguish the presence of AKI from pre-existing CKD.

## 4. Discussion

The current criteria for the diagnosis of AKI based on elevated levels of serum creatinine or BUN are often inadequate for the early detection of renal injury. Injury to the renal tubules may not be sufficiently severe to cause changes in the serum creatinine or BUN levels. Therefore, injury biomarkers for the detection of tubular damage, used in addition to the functional markers, may facilitate the early recognition of renal injury. Our study revealed the elevation of the urinary MIF level in APN patients with kidney infection, and urinary MIF serves as an injury biomarker of AKI in these patients.

In mice models of endotoxic shock or *E. coli* peritonitis, elevated serum MIF was detected, and MIF neutralizing antibodies protected the mice from lethal shock and sepsis [33, 34]. Calandra et al. have reported that streptococcal and staphylococcal exotoxin induced MIF secretion in macrophage, and anti-MIF antibody increases survival in mice model of exotoxin-induced shock [35]. Taken together, these findings indicate that MIF has an important role in bacterial infections. Several clinical studies have indicated that septic patients with high serum MIF levels appear to have a higher risk of mortality than patients with lower serum MIF levels [36–38]. In our investigation, however, the serum MIF level was not sufficient to detect the presence of AKI. We found that the urinary MIF level is a more sensitive indicator of kidney injury than the systemic MIF level, as suggested by Brown et al. [17, 18]. Previous studies have focused on the use of elevated urinary MIF levels to detect the presence of UTIs and to distinguish kidney infections from acute cystitis [23, 24]. In patient with normal renal function, consistent with previous study, our study revealed that elevated urinary MIF indicated the presence of APN. Our investigation further demonstrated that high urinary levels of MIF suggest the presence of AKI. In patients with elevated serum creatinine or BUN levels, it is a clinical puzzle to determine whether patients have AKI or pre-existing CKD. In subgroup analysis of patients with renal dysfunction on arrival, we revealed that the patients with AKI had an increase in urinary MIF compared to patients with pre-existing CKD. MIF has been reported to be an injury marker in kidney inflammatory disease, and consistently, our results revealed the utility of urinary MIF in determined AKI under infection. We, therefore, provide evidence supporting the ability of the urinary MIF level to identify patients with AKI and to discriminate AKI from CKD in patients with renal dysfunction.

Our study of APN patients also revealed increased urinary IL-1*β* levels in AKI patients. The urinary IL-1*β* level has been previously reported as a marker for APN [30]. IL-1*β* is secreted in biological fluids and thought to be a primary initiator of the inflammatory cascade during bacterial infection [39]. The levels of urinary IL-1*β* were correlated with the levels of urinary MIF in the present study. The regulatory role of MIF in IL-1*β* production has been demonstrated in previous studies, which demonstrated that MIF promotes inflammation through autocrine and paracrine effects to induce the production of IL-1*β* by nearby tissues or immune cells [8, 40]. Furthermore, elevated levels of MIF in the urine have been found in individuals with the progressive form of glomerulonephritis and those experiencing renal allograft rejections. All of these reports reinforce the role of MIF in renal damage [17, 18].

The urothelium contains a rich store of preformed MIF. During cystitis, MIF is upregulated in the bladder, released from the bladder, and detected in the urine as a potential marker for cystitis [24, 41]. Otukesh et al. have revealed an increase in urinary MIF in patient with APN compared to that in patient with cystitis, suggesting renal origin, in addition to cystic origin, for excretion of urine MIF [23]. In human glomerulonephritis, elevated concentrations of urinary MIF reflect the severity of renal injury and AKI. A significant correlation of urinary MIF and renal MIF implicates a renal origin for the excreted urine MIF during AKI [17]. Based on the study mentioned earlier, our investigation confirmed the applicability of urinary MIF in detecting APN-related AKI. The stepwise increase in urinary MIF may originate from both bladder and kidney, reflecting the extent of bacterial invasion. 

The urinary levels of MIF were correlated with the levels of KIM-1 but not with the urinary WBC count. KIM-1 is highly and specifically overexpressed by the proximal tubular cells under conditions of nephrotoxic AKI and is, therefore, a sensitive urinary biomarker to detect renal tubular injury [42, 43]. MIF is constitutively expressed in renal tubules in normal kidneys and released and then performs its biological function related to renal inflammatory disease [8]. The positive correlation between the urinary levels of MIF and KIM-1 suggests that renal tubules are one of the origins of urinary MIF. Renal tubular cells expressing TLRs contribute to the activation of the inflammatory response during ischemia-reperfusion injury in rat kidneys [44, 45]. TLR4 on renal epithelial cells activates the immune response and participates in the renal clearance of uropathogenic *E. coli* [46]. Additionally, our previous studies and those of others have shown that inhibition of MIF suppresses TLR4-induced inflammatory cytokine production via alternations in ERK, p38 mitogen-activated protein kinase, and NK-*κ*B activation [13, 14]. The relationship between increased MIF levels in patients with UTI-related renal inflammation and TLR4 is, therefore, speculative.

In summary, we found that the urinary MIF level is significantly elevated in AKI patients during kidney infection. The elevated level of MIF was significantly correlated with the urinary IL-1*β* and KIM-1 levels, which are indicative of injury to the renal tubules. These findings suggest that urinary MIF is a potential biomarker and that the measurement of the urinary MIF level may serve as a useful tool for recognizing nephrotoxicity in APN patients.

## Figures and Tables

**Table 1:** Demographic data and clinical characteristics of patients with APN.

Clinical variables	Number of cases (%)		
	Without AKI (*n* = 26)	With AKI (*n* = 13)	*P* value
Age (yrs)	66 ± 18	73 ± 10	0.204
Male	9 (35)	4 (31)	1.000
Comorbidity			
Diabetes mellitus	13 (50)	6 (46)	1.000
Hypertension	15 (58)	9 (69)	0.728
CKD (eGFR < 60 mL/min/1.73 m^2^)	7 (27)	6 (46)	0.290
Baseline eGFR^a^	79 ± 33	67 ± 46	0.322
Laboratory data			
White blood cell count (k/*μ*L)	12.7 ± 4.8	13.9 ± 8.4	0.565
Absolute neutrophil count (k/*μ*L)	10.8 ± 4.8	11.1 ± 7.5	0.858
C-reactive protein (mg/dL)	99 ± 107	115 ± 67	0.669
Creatinine (mg/dL)	1.4 ± 1.3	3.4 ± 2.0	0.003
Blood urea nitrogen (mg/dL)	28.9 ± 29.6	68.7 ± 36.2	0.001
Current eGFR^b^	78 ± 40	22 ± 15	<0.001
Urine MIF (ng/mg)^c^	4.2 ± 3.5	17.0 ± 13.2	0.004
Serum MIF (ng/mL)	259.6 ± 240.9	279.8 ± 248.1	0.810
Gram-negative bacteria^d^	24 (92)	12 (92)	1.000
Severity score			
REMS	7.5 ± 3.6	7.7 ± 2.8	0.893
RAPS	2.1 ± 2.0	3.2 ± 2.3	0.174

Categorical variables are expressed as a number (percentage), and continuous variables are expressed as the mean ± SD. AKI: acute kidney injury; CKD: chronic kidney disease; eGFR: estimated glomerular filtration rate; MIF: macrophage migration inhibitory factor; REMS: rapid emergency medicine score; RAPS: rapid acute physiology score.

^a^The baseline eGFR was estimated using the MDRD equation, and the units are mL/min/1.73 m^2^.

^b^The current eGFR was estimated by the MDRD equation using the serum creatinine level while the patient arrived at the emergency department.

^c^The concentrations of MIF in the urine were measured by ELISA and were normalized based on the urinary creatinine levels. We divided the urine levels of MIF by urine creatinine to measure the adjusted urine MIF (ng/mg).

^d^The bacterial pathogens of APN were identified and proven as Gram-negative bacteria.

**Table 2:** Microbiological analysis of 39 patients with APN.

Invaded pathogens^a^	Number of cases (%)	
	Without AKI (*n* = 26)	With AKI (*n* = 13)
Gram-negative bacteria	24 (92)	12 (92)
* E. coli *	17 (65)	10 (77)
* Proteus mirabilis *	2 (8)	0 (0)
* Klebsiella pneumoniae *	1 (4)	2 (15)
* Pseudomonas aeruginosa *	3 (12)	0 (0)
* Providencia stuartii *	1 (4)	0 (0)
Gram-positive bacteria	2 (8)	1 (8)
* Enterococcus* species	2 (8)	0 (0)
* Coagulase-negative staphylococcus *	0 (0)	1 (8)

Variables are expressed as a number (percentage). AKI: acute kidney injury.

^a^The bacterial pathogens of APN were identified and proven as shown later.

**Table 3:** Subgroup analysis of urinary MIF levels between APN patients with diabetes, renal dysfunction, or microbiological analysis.

Clinical characteristics of APN patients	Number of cases	Without AKI	With AKI	*P* value
Gram-negative bacteria^a^	36	4.4 ± 3.5	16.5 ± 13.8	0.011
* E. coli *	27	4.0 ± 3.4	15.2 ± 11.6	0.013
Diabetes mellitus	19	4.1 ± 4.1	15.2 ± 8.7	0.024
Renal dysfunction on arrival^b^	21	2.9 ± 2.4	17.0 ± 13.2	0.002

Urinary MIF levels (ng/mg) are expressed as the mean ± SD. AKI: acute kidney injury; eGFR: estimated glomerular filtration rate.

^a^The bacterial pathogens of APN were identified and proven as Gram-negative bacteria.

^b^The patients who presented with renal dysfunction (defined as eGFR < 60 mL/min/1.73 cm^2^) upon arrival at our emergency department were included.
